# Assessment of Avermectins-Induced Toxicity in Animals

**DOI:** 10.3390/ph15030332

**Published:** 2022-03-09

**Authors:** Muhammad Salman, Rao Zahid Abbas, Khalid Mehmood, Riaz Hussain, Sehar Shah, Mehwish Faheem, Tean Zaheer, Asghar Abbas, Bernardo Morales, Ina Aneva, José L. Martínez

**Affiliations:** 1 Department of Parasitology, University of Agriculture Faisalabad, Faisalabad 38000, Pakistan; msalmanhameed@gmail.com (M.S.); shahsehar12@gmail.com (S.S.); teanzaheer942@gmail.com (T.Z.); 2 Department of Clinical Medicine and Surgery, The Islamia University of Bahawalpur, Bahawalpur 63100, Pakistan; khalid.mehmood@iub.edu.pk; 3 Department of Pathology, The Islamia University of Bahawalpur, Bahawalpur 63100, Pakistan; driazhussain@yahoo.com; 4 Department of Zoology, Government College University Lahore, Lahore 54000, Pakistan; mehwishfaheem@gcu.edu.pk; 5 Faculty of Veterinary and Animal Sciences, Muhammad Nawaz Shareef University of Agriculture Multan, Multan 59300, Pakistan; abbasasghar255@gmail.com; 6 Department of Biology, Faculty of Chemistry and Biology, University of Santiago de Chile, Estación Central, Santiago 9160000, Chile; 7 Institute of Biodiversity and Ecosystem Research, Bulgarian Academy of Sciences, 1113 Sofia, Bulgaria; ina.aneva@abv.bg; 8 Vicerrectoria de Investigación, Desarrollo e Innovación, Universidad de Santiago de Chile, Estación Central, Santiago 9160000, Chile

**Keywords:** avermectins, parasite, toxicity, animals, safety

## Abstract

Macrocyclic lactones, particularly the avermectins, have completely revolutionized the approaches aimed at control of parasites. These avermectins are the most widely used anti-parasitic drugs in veterinary field with sales exceeding one billion US dollars annually. However, before clinical usage, their safety evaluation in the animals is a major critical factor that must be considered. Many studies have reported the negative effects of avermectins like ivermectin, abamectin, doramectin, and eprinomectin on the host animals. These harmful effects arise from avermectins targeting GABA and glutamate-gated chloride channels present both in the parasites and the host animals. In this review, various modes of avermectins action along with the negative effects on the host like nephrotoxicity, hepatotoxicity, neurotoxicity, reproductive toxicity, and endocrine disruption were discussed in detail. Furthermore, other important issues like ecotoxicity, drug resistance, and drug residues in milk associated with avermectins usage were also discussed, which need special attention.

## 1. Introduction

Parasitic organisms like helminths, insects, and arachnids pose a serious threat to the welfare of both humans and animals [1,2,3,4,5]. There exists a significant host-parasite specificity with the parasites getting their metabolic and physiological needs from their hosts [3]. They not only cause diseases, but also result in huge economic losses in terms of reduced productivity and retarded growth in their hosts, thus emphasizing the need for control of these parasites using novel approaches [6,7]. Basically, there are two approaches aimed at control of parasites including either the use of synthetic molecules or the use of natural products [3].

The chemical and structural diversity of natural products has always revolutionized the biological field with the discovery of new drugs [8]. For example, the discovery of avermectins, a group of macrocyclic lactones, in 1976 in Japan changed the concept of parasite control [9,10]. These avermectins are produced by fermentation of *Streptomyces avermitilis*, and possess broad-spectrum activities against insects, arachnids, and nematodes [11,12,13,14]. They are used extensively in the veterinary field and their annual sales exceeded one billion US dollars in past two decades [10]. One of the main reasons behind their extensive use is their availability in tablets/bolus, drench/syrup, and sometimes injection forms.

Avermectins, termed as endectocides, are recommended for use in many animal species like sheep, goat, cattle, horses, cats, and dogs for control of both internal and external parasites [15,16,17,18,19]. However, the assessment of possible drug toxicity in animals is an important aspect when using drugs for control of parasites, particularly in food animals. In April, 1999, the Safety Working Group of Veterinary International Conference on Harmonisation agreed that the evaluation of veterinary drugs for their safety in food animals is an important endpoint for toxicity studies [20].

Besides the veterinary use, avermectins also find a crucial role in human medicine. Among the avermectins family, ivermectin is currently the only drug which is licensed for use in humans [4]. It is used to treat health conditions like onchocerciasis, strongyloidiasis, lymphatic filariasis, and crusted scabies [10,21,22,23]. As far as the toxicity is concerned in humans, ivermectin damages the macrophages and interacts with liver cytochrome P450 enzymes, thus producing immunotoxicity and hepatotoxicity [24,25]. Other clinical signs observed in patients receiving ivermectin therapy may include pruritis, malaise, skin edema, hypotension, headache, and dyspnea [21].

Various studies have also indicated the avermectins to have toxic effects on animals [26,27,28,29]. For example, collie dogs experiencing severe illness presented to the clinic were found to be suffering from ivermectin toxicity [30]. Similarly, the therapeutic dose of ivermectin in adult rats was found to impair neurochemical and behavioural attitudes [17]. Other studies conducted on cows indicated therapeutic doses of ivermectin to induce hormonal changes, ultimately affecting the reproductive cycle of cows [31,32]. Other studies have also revealed the administration of avermectins even at therapeutic doses to cause reproductive, hepato-renal, sexual, and behavioural abnormalities in animals [17,33,34]. In mammals, these drugs are even more harmful at the juvenile stage as compared with the adult stage because juveniles are still undergoing the stages of physical development. Additionally, the metabolic rates of these drugs are different in juveniles as compared with adults, making the juveniles more prone to rapid toxicity [17]. Moreover, the molecular pathways leading to the idiosyncratic behaviour of avermectins are still ambiguous. In this review, the details of avermectins toxicity were described along with their possible modes of action.

## 2. Avermectins

**Figure 1:**
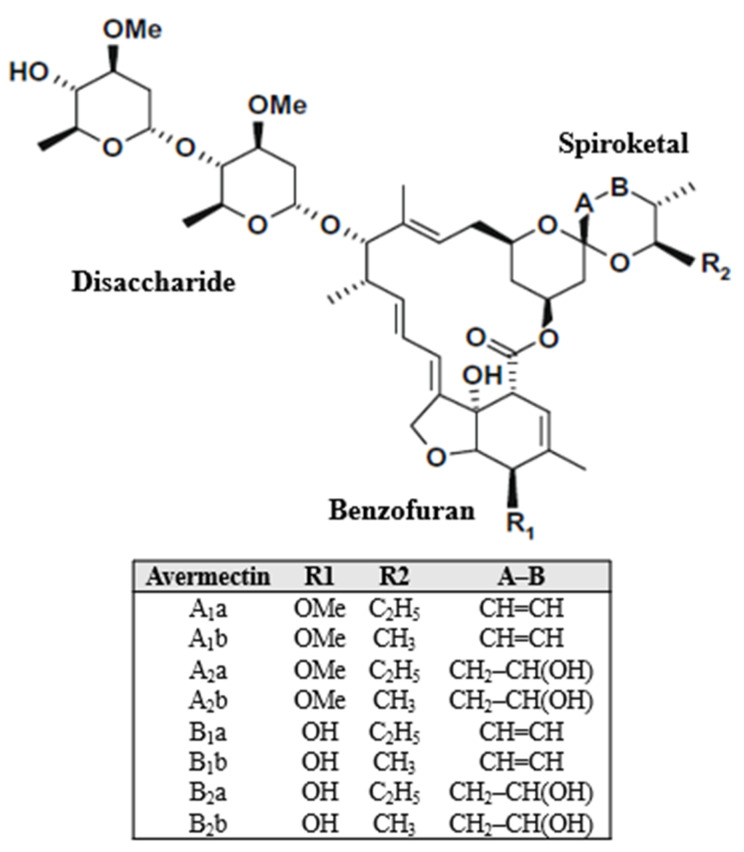
General structural outline of avermectins.

Avermectins are the group of 16 distinct chemical compounds that belong to the drug category of macrocyclic lactones and which have well established insecticidal, acaricidal, and nematicidal activities [11,12,13,14]. These are originally produced by soil dwelling gram-positive bacterium of group actinomycetes, *Streptomyces avermitilis*, through the process of fermentation [35,36,37,38]. Macrocyclic backbone is the main component of avermectins to which a hexahydrobenzofuran and a spiroketal agent are attached [11]. At the C-13 position, the avermectins possess a bisoleandrosyloxy group as the main identification point [11,13,36]. There are eight different components classified into two main groups A and B (A1_a_, A2_a_, A1_b_, A2_b_, B1_a_, B2_a_, B1_b_, and B2_b_) which are yielded by bacterium *Streptomyces avermitilis*. The A and B components vary only with respect to the presence of either methoxy or hydroxy groups at the C-5 position respectively. The general structure of avermectins described by various scientists is elaborated by Figure 1 [12,35,39].

**Figure 2:**
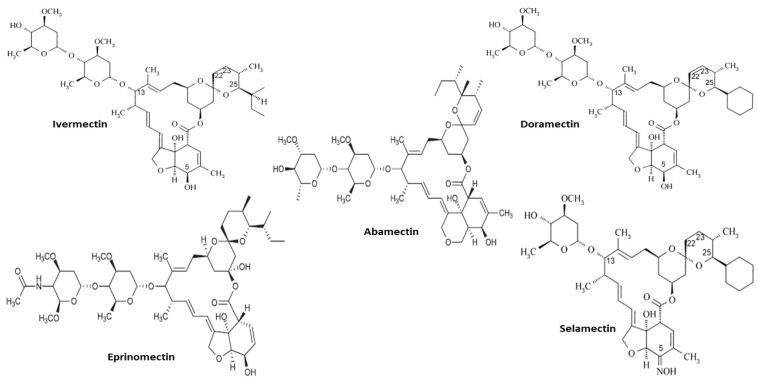
Chemical structure of various avermectins.

Examples of avermectins include ivermectin, abamectin, doramectin, eprinomectin, and selamectin [11,13,15,23,36,38,40]. The chemical structures of these compounds as taken from various sources [15,40] are shown in Figure 2.

### 2.1. Mode of Avermectins Action against Parasites

**Figure 3:**
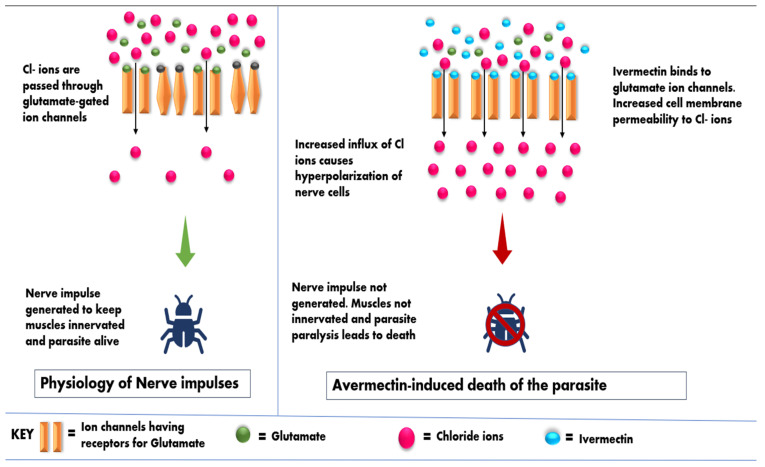
Mode of action of avermectins.

Avermectins, regarded as potential neurotoxins, target the gamma aminobutyric acid (GABA) receptors and the glutamate gated chloride ion channels (GluCl) which are concerned with neurotransmission in parasites [10,17,41]. GABA, a neurotransmitter, causes opening of the chloride ion channels of the organism resulting in the influx of chloride ions. Avermectins act as agonists of chloride channels and cause changes in permeability of these channels, thus resulting in neurotransmission disturbance [13,36,42,43,44]. This disturbance induces neuronal membrane hyperpolarization, paralysis, and ultimately the death of the parasite. The GABA receptors in the mammals are found only in the brain, which is shielded by the blood-brain barrier. Avermectins cannot cross this protective barrier, thus making the avermectins relatively safe in mammals [10,13,17,45]. This mode of action is briefly summarized in Figure 3.

### 2.2. Toxicity Studies

The avermectins are potent antiparasitic drugs which may pose a serious toxicity threat to animals. By damaging the various organs of the body, these may even lead to death of animals, as seen in various cases [27,46,47,48]. These damaging effects are seen in dose-dependent and dose-time dependent manners [49,50]. The reason is that avermectins are lipophilic and, hence, tend to accumulate in fatty tissues and the liver where they induce oxidative stress leading to tissue damage through lipid peroxidation [51]. The other important factor is the breed susceptibility. In different animals, the same doses may be damaging. Additionally, some animals like dogs and rats are deficient in P-glycoprotein, which acts as an efflux pump against avermectins, thus increasing their vulnerability to the negative effects of avermectins [30,52]. In toxicity analysis, a combined evaluation of various biochemical parameters provides better identification of the organ being damaged by the drug under investigation. A detailed review of various toxicity studies is described below.

### 2.3. Nephrotoxicity

The evaluation of serum creatinine and the blood urea levels post-administration of drugs provides a good overview for nephrotoxicity analysis [46]. Various studies conducted on avermectins (mainly the ivermectin and the abamectin) have proven these drugs to induce nephrotoxicity in many animals like mice, bats, rabbits, and rats [28,34,53,54,55,56]. The main molecular mechanism through which avermectins exert their nephrotoxic effect is the lipid peroxidation which results from the action of reactive oxygen species [51]. This oxidative damage results in histopathological changes like interstitial nephritis, glomerular damage, interstitial infiltration areas of round cells, and tubular necrosis as well as elevated levels of serum creatinine, urea, and the uric acid in the blood [46,57,58]. For example, ivermectin causes nephrotoxicity through elevated levels of creatinine and decreased protein synthesis and glucose levels in pigs and dogs [59,60]. Various other reports of avermectins causing nephrotoxicity are summarized in the Table 1.

### 2.4. Hepatotoxicity

The liver is the main organ that catabolizes and neutralizes most toxins and drugs present in the body [66]. These drugs or toxins may induce hepatic injury, which can escalate into complete hepatic failure and even death of the animal may also occur [67]. Avermectins are highly lipophilic with the highest concentration found in the liver of animals post-administration regardless of the administration route [68,69,70]. They cause hepatotoxicity by affecting the liver enzymes, altering the mitochondrial bioenergetics of hepatocytes, inducing oxidative stress, and enhancing the autophagy in the liver tissues of the treated animals [65,66,71,72,73,74]. Various histopathological changes are also observed like dilated blood vessels, infiltration of leucocytes, and hepatocytes degeneration [75]. As a repairing process, autophagy clears the damaged organelles and proteins produced as a result of hepatocytes damage. For example, avermectins administration in pigeons at different concentrations induced hepatotoxicity and increased the apoptosis in a dose dependent manner [74]. Similarly, other hepatotoxic reports of avermectins are compiled in Table 2.

### 2.5. Neurotoxicity

Generally, the use of avermectins in animals has a wide safety margin as far as the nervous system is concerned [77,78]. This is due to the P-glycoprotein associated with the blood-brain barrier that prevents the avermectins from exerting their neurotoxic effects by inhibiting their penetration in the brain [78,79]. P-glycoprotein is responsible for multi-drug resistance and uses avermectins as substrate [77,80]. Thus, the P-glycoprotein expression is linked with the neurotoxicity of avermectins in animals like rats and dogs, which are somewhat deficient in P-glycoprotein [30,81]. The avermectins exhibit nervous effects by damaging the cerebral cortex and inducing diffused cerebellar dysfunction [79]. They do so by modulating the binding of GABA and benzodiazepine receptors, affecting the chloride channels and targeting the Cys-loop receptors of the mammalian brain [39,82,83]. Various signs observed in neurotoxicity studies are depression, tremors, salivation, ataxia, convulsions, mydriasis, coma, and ultimately death in animals like dogs, cattle, and lions, etc. [77,78,79,84]. Neurotoxic effects produced by various avermectins are described in Table 3.

### 2.6. Reproductive Toxicity

There are reports of avermectins negatively affecting the reproductive system of the animals [34,46]. As discussed earlier, avermectins induce neurotoxicity by damaging the brain, which is responsible for production of reproductive hormones; therefore, they indirectly affect the reproductive system of animals as well [63,89]. In males, avermectins cause testicular damage, thereby affecting the sperm count as well as the sperm motility [63,90]. The avermectins, by crossing the blood-testes barrier, reach the germ cells of the seminiferous tubules, causing a reduced meiotic index that ultimately results in decreased sperm count [63,91]. The targeting of the brain and the testes by avermectins results in sexual behavioural changes as well as impaired hormones production [92,93]. Likewise, avermectins also target the female reproductive organs, producing deleterious effects like degenerated and hemorrhagic reproductive organs, degenerated ova, and atritic follicles [62]. They have the potential of crossing the placental barrier and are also passed in milk during lactation and produce developmental anomalies in offspring at high doses [94,95]. Various reports of avermectins causing reproductive toxicity are summarized in Table 4.

### 2.7. Endocrine Disruption

The organisms administered sub-lethal doses of a drug may experience different side effects including the endocrine disruption [96]. These are the endocrine glands which produce hormones responsible for metabolism, growth, and development of cells. Thus, any disruption in the endocrine system will have a negative impact on the organism [97]. At present, there is limited research available describing the role of avermectins as endocrine disruptors. Additionally, there are different gaps regarding mammalian endocrinal toxicology which are not properly addressed [98]. Among the avermectins, ivermectin and abamectin are investigated as endocrine disruptors. Sexual maturation in Holstein heifers was advanced by 3.5 months following continuous ivermectin administration from birth till puberty. This early maturity may be associated with the elevated levels of insulin-like growth factor and luteinizing hormone [31]. In Baladi cows, the injection of a therapeutic dose (0.2mg/Kg) of ivermectin one day post-parturition caused 3 months delay in estrous. It caused disturbances in the levels of luteinizing hormone, follicle-stimulating hormone, cortisol, estradiol, progesterone, and prolactin [32]. Similarly, ivermectin was also shown to suppress the sexual behavior in estradiol treated female rats at therapeutic dose [99]. In another study where the male albino rats were exposed to sublethal dose of abamectin, significant alterations in sex hormones as well as the thyroid hormones were observed [97,100,101]. Likewise in humans, abamectin is placed under the category which is more likely to cause endocrine disruption according to the joint UK-German document [102].

**Figure 4:**
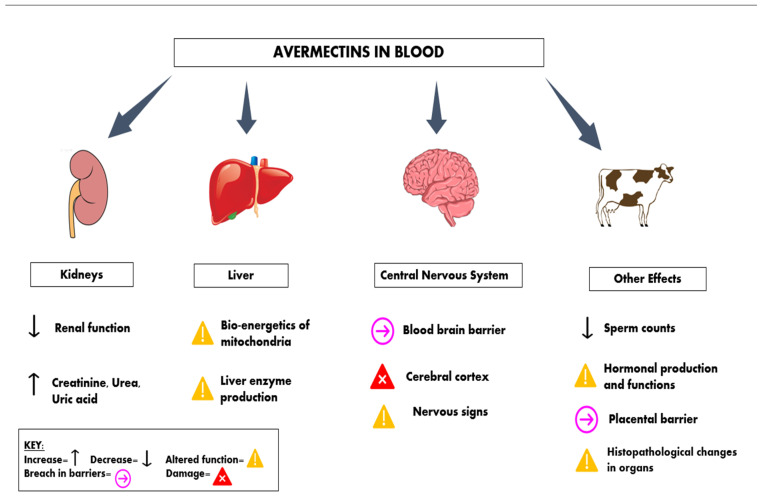
Description of avermectins-induced toxicity.

The toxic effects of avermectins in animals discussed above can be briefly described, as in Figure 4.

## 3. Missing Gaps and the Future Perspective

### 3.1. Milk Residues

The lipophilic drugs in plasma easily cross the epithelial barrier of mammary glands and concentrate in the milk. The same is true with the avermectins which are highly lipophilic in nature and, hence, diffuse readily in milk [23,103]. This lipophilicity can be estimated from concentration ratio of drugs in milk vs. plasma. The greater the value, the higher the lipophilicity of the drug. In various reports, ivermectin and abamectin were shown to have concentration values close to 1 and 0.2 respectively in the experimental animals, showing abamectin to be relatively safe in view of milk residues [104,105]. The milk with drug residues is fit neither for human consumption nor for animals’ offspring. So, there is a need for either the discovery of new, safe drugs or the development of a new administration technique that minimizes the milk residues and the milk-rejection period.

### 3.2. Resistance

Avermectins have been used in animals as mass drug administration strategy (MDA) with the purposes of treatment and prophylaxis from parasitic diseases. However, this extensive application of the drugs has led to rapid selection and resistance in the parasites which can survive the drug administration [37]. Various studies have shown the resistance to be associated with genetic mutations but the main mechanism of development of resistance is yet not known [37,106]. Some of the techniques like genetic crossing and genome-wide sequencing may help us better understand the mechanism of resistance development towards avermectins [107,108,109]. Apart from understanding the resistance development mechanism, there is need to develop integrated approaches using various options for control of parasites [37,106].

### 3.3. Ecotoxicity

Administration of avermectins in animals results in their excretion of faeces, thus posing an impact on the biological processes of nature [110]. The non-specificity of avermectins in their action leads them to exert their deleterious effect not only on the parasites but also on the non-parasitic species found in the ecosystem like dung beetles. These non-parasitic environmental organisms may have an important role in food webs like nutrient recycling in the environment and organic matter decomposition [110]. The sustained slow-release strategy for avermectins poses an even greater threat to the ecosystem [111]. The avermectins are somewhat resistant to environmental degradation and, thus, tend to accumulate in the environment [112]. For example, there are reports of a direct relationship between the dung beetle population in the field and the concentration of avermectins in dung [13,113]. It is important to highlight the potential effects of un-controlled and over the label use of Avermectins in animals and humans. The magnitude of resistance may further be amplified due to the residues of avermectin drugs in the environment. Therefore, there is a need to adopt some comprehensive strategy for the use of avermectins in animals to protect the environment from their toxic effects.

## 4. Materials and Methods

We performed a systematic review of the scientific literature using the Web of Science, PubMed, and Google Scholar databases through multiple combinations of Avermectins terms with “Toxicity”, “Animals”, “Parasite”, and “Safety”. We limited the search to studies in various types of toxicity (Nephrotoxicity, Hepatotoxicity, Neurotoxicity, Reproductive Toxicity). We obtained over 500 articles that were analyzed and subsequently those that corresponded to the objectives of this study were selected. Following this criterion, we chose and used 113 articles as a reference for this review.

## 5. Conclusions

The avermectins, owing to their broad spectrum of action, find a crucial role in parasitic control practices with huge sales of these products throughout the world. However, the side effects linked with their usage puts emphasis on a comprehensive approach towards understanding all the aspects associated with them. Due to their non-target specificity, these act both on the parasites as well as the host animals to which they are administered. Nephrotoxicity, hepatotoxicity, neurotoxicity, and reproductive toxicity are the main side-effects produced by them in the host animals. Apart from these effects, these compounds also pose a major threat to the ecosystem and the food web with additional risk of resistance development in the parasites. So, there is need for research focusing on the above-mentioned issues to overcome these major constraints of avermectins usage in animals.

## Figures and Tables

**Table 1:** Nephrotoxic avermectins along with dose, animal, and outcome.

Drug	Dose	Animal	Outcome	Reference
Ivermectin	One drop 1% topically	Bats	Proliferative glomerulonephritis, tubular necrosis	[53]
	2 mg/Kg BW (2 injections with 15 days interval) subcutaneously	Goats	Glomerular necrosis, degeneration of tubular epithelium, necrosis of capillary tuft, elevated blood levels of uric acid, urea, creatinine, and glucose	[61]
	Weekly 0.5 mg/Kg BW for 8 weeks subcutaneously	Rabbit	Subcapsular tubules vacuolation, glomerular atrophy, elevated serum creatinine	[54]
	Weekly 0.4 mg/Kg BW for 4 weeks subcutaneously	Rabbit	Congested blood vessels, tubular degeneration, desquamation and necrosis of tubular epithelium, hyaline casts, leucocytic infiltration, and cystic dilatation of tubules	[62]
	6.5 mg/Kg BW (1/5th of LD_50_) single dose orally	Mice	Elevated levels of creatinine and urea, renal edema, necrosis and karyorrhexis of tubular epithelium, Bowman’s space narrowing	[28]
Abamectin	0.181 mg/Kg BW (1/100 of LD_50_) for 30 days orally	Rats	Elevated levels of creatinine and uric acid, nephritis	[57]
	2.18 mg/Kg BW (1/4 of LD_50_) single dose orally	Rats	Elevated levels of creatinine, uric acid, and urea	[63]
	0.44 mg/Kg BW (1/20 of LD_50_) for 4 weeks orally	Rats	Elevated levels of creatinine, uric acid and urea, induced oxidative stress, necrosis, congestion, edema and nephritis	[64]
	10 mg/Kg BW for 6 weeks orally	Rats	Elevated levels of creatinine, uric acid and urea, glomerular and tubular necrosis, hemorrhages in cortex	[58]
	30 mg/Kg BW for 30 days orally	Rats	Elevated levels of creatinine and urea, oxidative stress	[51]
	0.1 mg/Kg BW for 15 days Intraperitoneal	Rats	Elevated levels of creatinine and urea, renal degeneration, congested blood vessels and renal casts	[55]
	2 mg/Kg BW (1/100 of LD_50_) for 5 days, Oral	Rats	Elevated levels of creatinine and urea, edema, hemorrhages, mononuclear cell penetration, glomerular atrophy and tubular necrosis	[56]
Avermectin 1a	20 mg/Kg feed for 60 days, Oral	Pigeons	Reduced cytochrome P450 concentration, Tubular swelling, vascular degeneration	[65]

**Table 2:** Hepatotoxic avermectins along with dose, animal, and Outcome.

Drug	Dose	Animal	Outcome	Reference
Abamectin	5 mg/Kg BW	Rats	↑Serum AST, ↑serum nitric oxide (NO)	[71]
	10µM	Rat	↓Liver mitochondrial respiration, inhibition of ATP synthesis	[66]
	2.13 mg/day per animal orally for 28 days	Rats	↑Glucose, ↑ASAT, ↑ALAT, Histopathological changes of liver	[75]
	10 mg/Kg BW orally	Rats	↑ALT, ↑AST, ↑ acid phosphatase (AP), ↑total protein, ↑albumin	[58]
	0.283 nM Inhibition constant	In-vitro goat liver	Carbonic anhydrase inhibition	[40]
	0.4 mg/kg SC	Calves	Liver swollen	[29]
Avermectin 1a	20 mg/Kg feed	Pigeons	Inhibition of cytochrome P450 enzyme	[65]
Avermectin B1a	20 mg/kg diet	Pigeons	Chromatin aggregation, mitochondrial damage	[73]
Doramectin	0.153 nM Inhibition constant	In-vitro goat liver	Carbonic anhydrase inhibition	[40]
Eprinomectin	0.232 nM Inhibition constant	In-vitro goat liver	Carbonic anhydrase inhibition	[40]
Ivermectin	50 mg/Kg Single dose LD_50_	Rats	Congested and haemorrhagic liver with centrilobar necrosis	[76]

**Table 3:** Neurotoxic avermectins along with dose, animal and outcome.

Drug	Dose	Animal	Outcome	Reference
Avermectin	20 mg/Kg diet	Pigeon	Increased expression of inflammatory factors, histological changes in cerebellum, cerebrum, and optic lobe	[85]
Avermectin1a	20 mg/Kg diet	Pigeon	Oxidative damage shown in brain and serum	[50]
Avermectin B1	120–200 µg/Kg	Murray Grey cattle	Incoordination, swaying gait, salivation, lingual paralysis and blindness	[86]
Abamectin	6 mg/Kg orally	Rats	Lowered weight of brain, decreased splay reflex, reduced motor activity	[87]
	30 mg/Kg orally	Rats	Changes in antioxidant defense markers of brain	[51]
Ivermectin	120 µg/Kg	Dog	Ataxia, mydriasis, hypersalivation	[88]
	0.8 mg/Kg subcutaneously for 8 weeks	Rabbits	Meningitis and brain degeneration	[62]
	1 mg/Kg subcutaneously	Rats	Increased serotonergic and dopaminergic system activity in association with stress	[17]
Doramectin	200 µg/Kg Subcutaneous	Border collie Dog	Ataxia, fever, tachypnoea, head pressing, hypersalivation, lack of menace response, and blindness	[26]
	0.2–0.5 mg/Kg plus horse carcass treated with doramectin	Lion	Ataxia, mydriasis, hallucinations, and death	[27]

**Table 4:** Reprotoxic avermectins along with dose, animal, and outcome.

Drug	Dose	Animal	Outcome	Reference
Abamectin	2.175 mg/Kg orally	Male Rats	↑WBCs count, ↓RBCs count, ↓haemoglobin, altered serum enzymes levels, reduced sperm count and motility	[63]
	10 mg/Kg orally once a week for 210 days	Male Rats	Decreased fertility, reduced number of offspring, histopathological changes in testes, degeneration of spermatogonia cells	[46]
	10 mg/Kg of BW orally	Male Rats	Intratubular edema in testes, degenerated and reduced number of spermatozoa	[58]
Doramectin	0.3 mg/Kg	Male Rats	Impaired sexual behaviour	[79]
	0.2 mg/Kg subcutaneously	Male Rats	Apoptosis of cells, focal degeneration areas in testes, necrotic spermatocytes, and decreased Sertoli cells count	[34]
Ivermectin	200 µg/Kg subcutaneously	Pregnant Cows	Transfer of drug in milk and colostrum, Accumulation of drug in calf plasma	[95]
	0.4 mg/Kg subcutaneously	Rabbits	Thickened testicular capsule, testicular edema, degenerated spermatogenic cells, atritic follicles and degenerated ova, desquamation of uterus glands	[62]
	0.2 mg/Kg subcutaneously	Male Rats	Apoptosis of cells, focal degeneration areas in testes, necrotic spermatocytes, and decreased Sertoli cells count	[34]

## Data Availability

Data are contained within the article.
